# Superinfected endometrioma, ultrasound guided drainage

**DOI:** 10.1016/j.radcr.2022.04.027

**Published:** 2022-05-11

**Authors:** Eric T. Wei, Amir-Ali Mahmoud, Kiyon Naser-Tavakolian, Dr. Lok Yun Sung

**Affiliations:** a Stony Brook University Hospital, Department of Radiology, 101 Nicolls Rd level 4, Stony Brook, NY 11794, USA; b Stony Brook Renaissance School of Medicine, 100 Nicolls Rd, Stony Brook, NY 11794, USA

**Keywords:** Infected endometriomas, Percutaneous drainage, Adhesions, Tubo-ovarian abscess

## Abstract

Infected endometriomas are rarely described in the literature with most cases being managed laparoscopically or open laparotomy. We present an infected endometrioma in a 48-year-old female with a history of extensive peritoneal adhesions in the setting of a contralateral tubo-ovarian abscess that was unresponsive to antibiotic therapy. Initially, the tubo-ovarian abscess was percutaneously drained, however, the patient did not clinically improve. The suspected infected endometrioma was then percutaneously drained which then led to clinical improvement. Typically, endometriomas are managed laparoscopically chiefly due to the risk of content spillage into the peritoneum, however, the case presented demonstrated that an ultrasound-guided transabdominal approach drainage can be feasible in a surgically complicated patient who was unresponsive to antibiotics in which a percutaneous approach was favored rather than a surgical approach.

## Case report

**Fig. 1:**
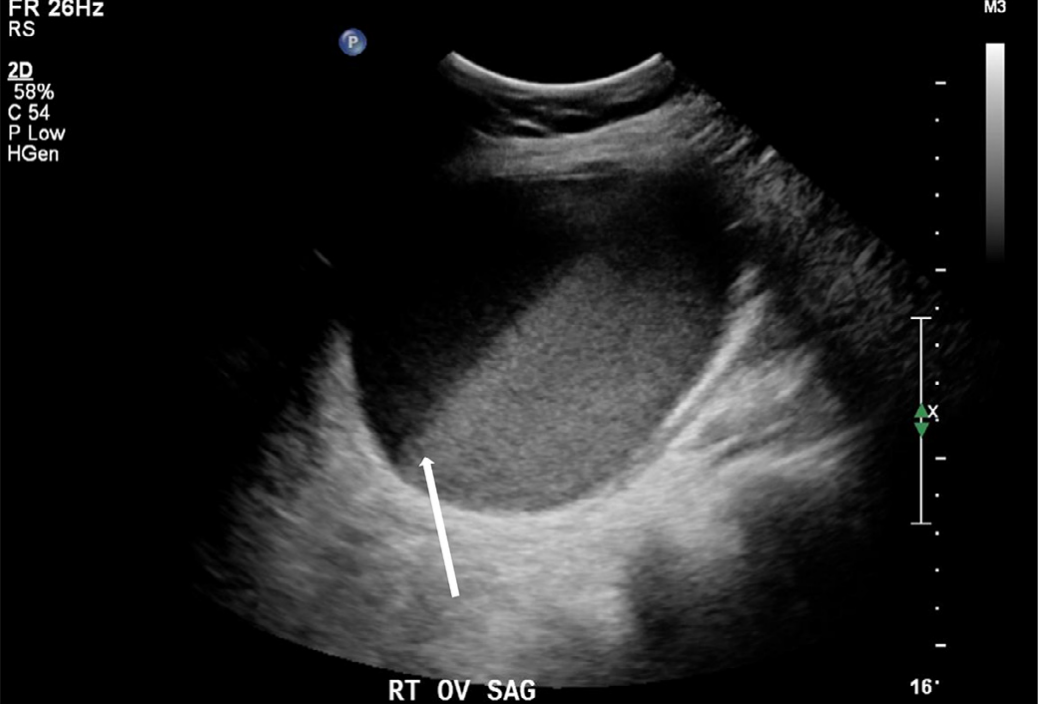
Transabdominal view of the right pelvis demonstrates target endometrioma in the right adnexa with evidence of a fluid-fluid layer (white arrow).

**Fig. 2:**
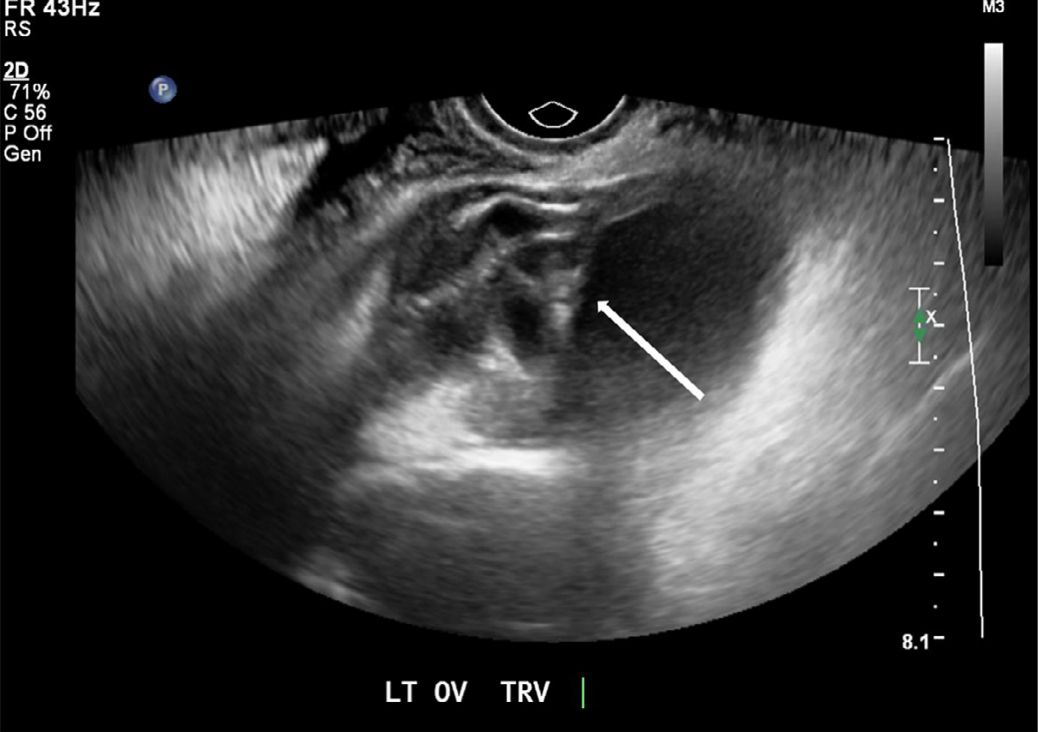
Initial transvaginal view demonstrates dilated tubular structure with multiseptated appearance in the left adnexa concerning for tubo-ovarian abscess (white arrow).

**Fig. 3:**
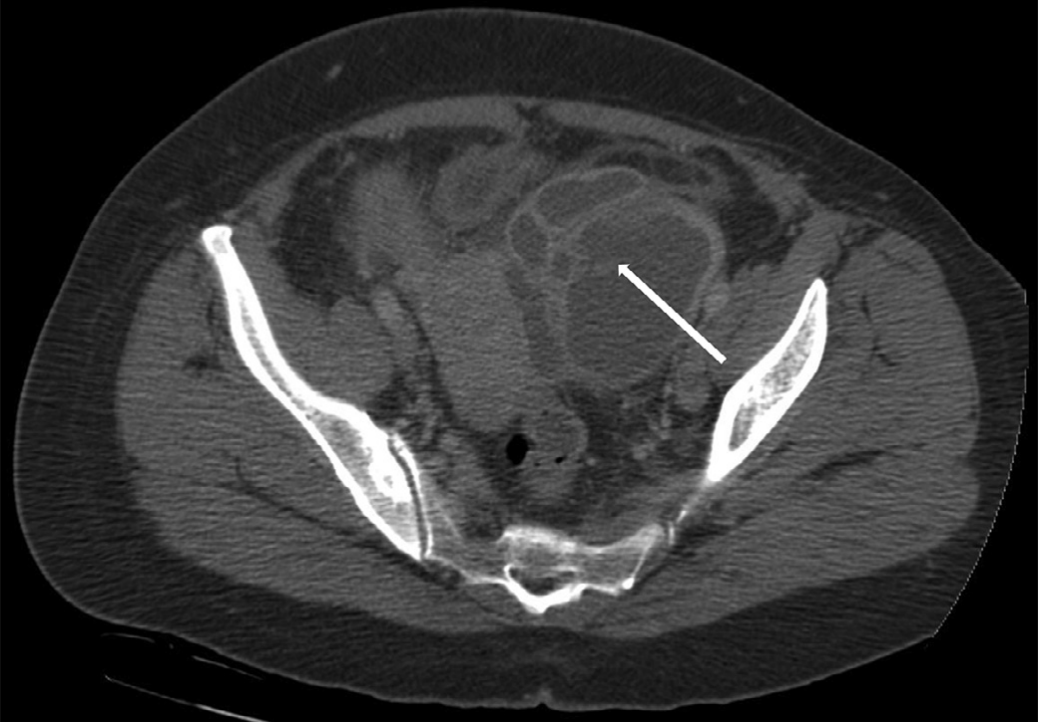
NECT of the pelvis demonstrates a tubular appearing structure with multiseptated appearance in the left adnexa (white arrow) with thickened walls and mild adjacent fat stranding concerning for a tubo-ovarian abscess.

**Fig. 4:**
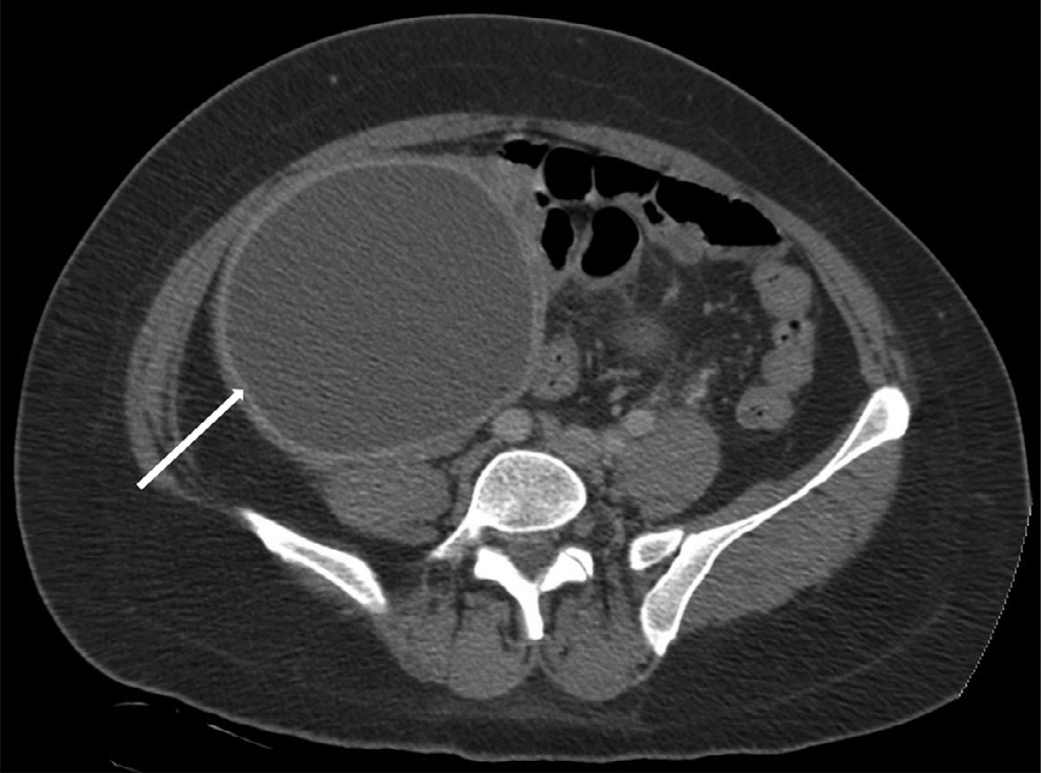
NECT of the pelvis demonstrates a large fluid containing structure in the right adnexa (white arrow) that corresponds to the pelvic ultrasound (Fig. 1) that was compatible with an endometrioma.

A 48-year-old female G0P0, newly diagnosed diabetes, chronic infertility, LMP 1 month prior to presentation with remote history of peritoneal pseudocyst status post exploratory laparotomy with extensive lysis of adhesions and drainage, remote history of pelvic inflammatory disease, and self-reported ovarian cysts, presented to the Emergency department with 5 days of lower abdominal pain associated with dysuria, hematuria, fevers, chills, and nausea. Vitals on presentation were temperature 39.5 C, heart rate 107 beats per minute, blood pressure 134/69, respiratory rate 15 per minute, pulse oximeter 97% on room air. Her initial laboratory values demonstrated a WBC count of 15.8 K/uL. Examination demonstrated soft abdomen with mildly diffuse tenderness of the abdomen, yellow purulent discharge at the cervix, bilateral adnexal fullness with left adnexal tenderness. Initial pelvic ultrasound (Figs. 1 and 2) and CT abdomen and pelvis (Figs. 3 and 4) demonstrated a dilated left salpinx with thickened enhancing walls and luminal attenuation concerning for tubo-ovarian abscess, and a large right adnexal layering lesion compatible with an endometrioma. The patient was started on IV cefoxitin, doxycycline, and flagyl. Gonococcal, chlamydia, RPR, and HIV panel were negative. The patients WBC continued to elevate to 16.5 K/uL, and continued to be febrile. Interventional radiology was consulted by Gynecology for percutaneous drainage of the left tubo-ovarian abscess.

Under moderate sedation, an 18-gauge needle was advanced via a left transabdominal approach into the left tuboovarian abscess under CT guidance. Over a series of exchanges, an 8.5-French multiside hole drainage catheter was placed. One hundred sixty mL of purulent fluid was aspirated which later grew *Escherichia Coli.*

Despite drainage of the left tuboovarian abscess, the patient continued to be febrile with elevated WBC to 17.3 K/uL despite IV antibiotics of 4 days. Antibiotic coverage was broadened to vancomycin and ertapenem for concern of MRSA and ESBL. Concern was then refocused for possible superinfected endometrioma, however, given the patient's history of significant pelvic adhesions with extensive lysis of adhesions, gynecology evaluated that the patient would likely require laparotomy for removal of the endometrioma, risking injury to surrounding organs and thus favoring percutaneous drainage with Interventional Radiology.

**Fig. 5:**
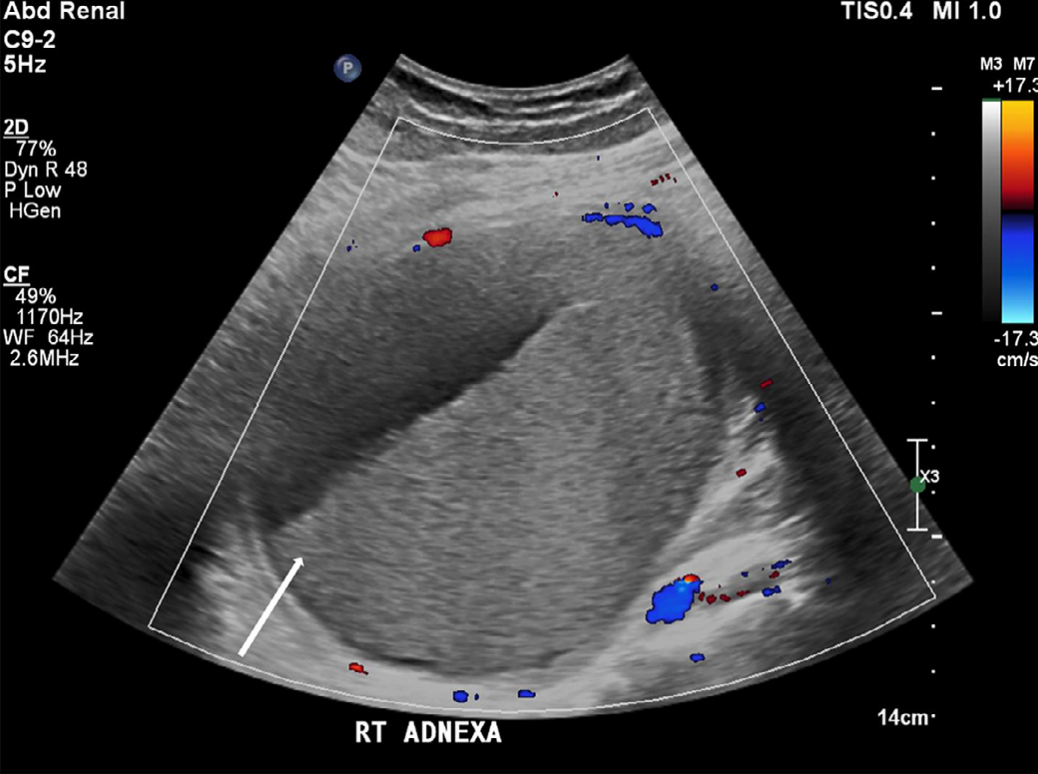
Transabdominal ultrasound of the pelvis with color flow with mildly increased peripheral vascularity. Again seen is fluid fluid layer within the endometrioma (white arrow).

**Fig. 6:**
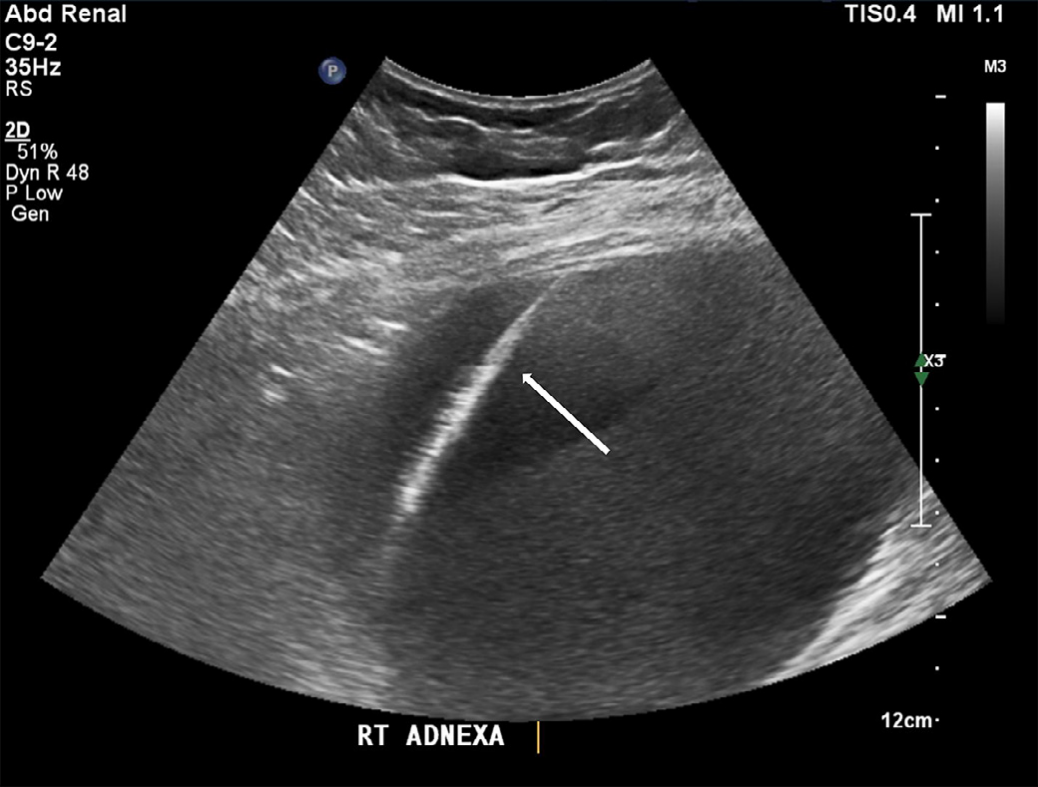
Transabdominal ultrasound of the pelvis with percutaneous catheter placement of the right adnexa demonstrates a fluid fluid with wire placement (arrow).

Under local sedation, a 21-gauge needle was placed into the right adnexal lesion under ultrasound guidance (Figs. 5 and 6) from an anterolateral transabdominal approach. Upon aspiration, cloudy serous fluid with pungent odor was observed. The needle was exchanged over a wire, and placement was confirmed with CT. Approximately 620 mL of chocolate brown purulent fluid was aspirated which later grew *Escherichia Coli* and *Bacteroides Thetaiotamicron.*

Following drainage of the right endometrioma, the patient's fevers subsided, and her white count normalized. Both susceptibility cultures demonstrated pansensitive *E. Coli* and antibiotics were narrowed to oral levofloxacin and metronidazole at time of discharge for total 21-day course. The patient was discharged on post procedure day 4 with bilateral drains in place for outpatient follow up with infectious disease and iterventional radiology. Repeat drainage check demonstrated collapse of the pelvic abscess cavity and the catheter was removed without complication.

## Discussion

Endometriosis occurs when there is presence of endometrial glands and tissue outside the uterine cavity thought to occur from peritoneal extension through retrograde menstruation [2]. Ovarian endometriomas are cystic masses formed by recurrent ectopic bleeding which result in thick brown fluid that is commonly referred to as “chocolate cysts” [3]. It has been conjectured that endometriomas serve as medium for bacterial growth and are susceptible to bacterial invasion through either direct infection (penetration or surgery), ascending route through the vagina or cervix, hematogenous spread, lymphatic spread, or direct spread from colonic wall [4]. Kubota et al [1]. also found that patients with endometriomas had a significantly higher frequency of developing tuboovarian abscesses In our case, both the tuboovarian abscess and the endometrioma cultured *Escherichia Coli.,* which can suggest an ascending infection, although Khan et al suggested that women with history of endometriosis appeared to have more contaminated menstrual blood with *E. Coli*. when compared to control women. Whether the mechanism is from translocation of E. Coli. from the gut enterocytes through to the pelvis or from contamination of menstrual blood after migration from the vagina to the uterine cavity has yet to be elucidated [5].

Management of large endometriomas typically involves laparoscopic surgical removal. Aspiration alone is discouraged given the high recurrence rate, and sclerotherapy has also been described, albeit with a relatively decreased rate of recurrence compared to that of aspiration alone. In contrast, our case differs due to the presence of infection, with the goal of treating the infection, rather than the endometrioma. To date, there is only one reported case in the literature of a percutaneous drainage of an infected endometrioma by Cornman Homonoff et al in which a patient with an infected endometrioma presumed to be secondary to recent hysterosalpingography was treated successfully [1].

While there is no established standard of care for infected endometriomas, management typically is the same as noninfected endometriomas. First line is laparoscopic cystectomy, followed by oophorectomy if that fails [1]. Ultrasound guided drainage of endometriomas has been shown to be a feasible treatment, however this technique can lead to complications. Recurrence of endometriomas following ultrasound guided aspiration occurred at a rate of 28 to 100% across four different studies [6]. While medical treatment with in situ injection of tetracycline, ethanol, methotrexate reduced recurrence, the risk was not fully eliminated. Percutaneous drainage also imposes the risk of infections, and aspirated contents may spill into the peritoneum causing adhesions which can lead to infertility and chronic pelvic pain [6]. Despite its risk of complications percutaneous drainage may be indicated in poor surgical candidates, such as in the case in our patient who previously had lysis of adhesions, or those with recurrence who have previously undergone laparoscopic surgery. It is important to note that a transvaginal approach can also be considered in practices that have the appropriate equipment and set up and may have more favorable outcomes in terms of patient comfort post procedurally. Given the size, anterior position, and equipment, transabdominal approach was favored.

In the presented case, the goal was not to treat and remove the endometrioma, but rather to treat the infection, which was accomplished through percutaneous drainage. The benefits of percutaneous drainage in this case, we believe, outweigh the risk of laparoscopic approach given the patients history of remote pelvic inflammatory disease and adhesive disease and the likelihood of a technically difficult surgical procedure.

## Conclusion

We report a case of a successful ultrasound guided percutaneous drainage of a superinfected endometrioma in the setting of a contralateral tuboovarian abscess. More follow up is needed to determine the likelihood of recurrence of the endometrioma and chances of reinfection.

## Patient consent statement

Informed consent was obtained from this patient prior to submitting this manuscript.
